# Immune Protection of SIV Challenge by PD-1 Blockade During Vaccination in Rhesus Monkeys

**DOI:** 10.3389/fimmu.2018.02415

**Published:** 2018-10-23

**Authors:** Enxiang Pan, Fengling Feng, Pingchao Li, Qing Yang, Xiuchang Ma, Chunxiu Wu, Jin Zhao, Hongbin Yan, Rulei Chen, Ling Chen, Caijun Sun

**Affiliations:** ^1^State Key Laboratory of Respiratory Disease, Guangzhou Institutes of Biomedicine and Health, Chinese Academy of Sciences, Guangzhou, China; ^2^School of Public Health (Shenzhen), Sun Yat-sen University, Guangdong, China; ^3^University of Chinese Academy of Sciences, Beijing, China; ^4^School of Life Sciences, University of Science and Technology of China, Hefei, China; ^5^Genor Biopharma Co. Ltd., Shanghai, China; ^6^The Guangzhou 8th People's Hospital & The First Affiliated Hospital of Guangzhou Medical University, Guangzhou, China

**Keywords:** PD-1 blockade, HIV vaccine, CTL, NHP model, SIV challenge

## Abstract

Though immune correlates for protection are still under investigation, potent cytotoxic T lymphocyte responses are desirable for an ideal HIV-1 vaccine. PD-1 blockade enhances SIV-specific CD8+ T cells. However, little information has been reported about how it affects the immunogenicity and protection of prophylactic SIV vaccines in nonhuman primates. Here, we show that PD-1 blockade during vaccination substantially improved protective efficacy in SIV challenged macaques. The PD-1 pathway was blocked using a monoclonal antibody specific to human PD-1. Administration of this antibody effectively augmented and sustained vaccine-induced SIV-specific T cell responses for more than 42 weeks after first immunization in rhesus monkeys, as compared with SIV vaccination only. Importantly, after intrarectally repeated low-dosage challenge with highly pathogenic SIVmac239, monkeys with PD-1 blockade during vaccination achieved full protection against incremental viral doses of up to 50,000 TICD_50_. These findings highlight the importance of PD-1 blockade during vaccination for the development of HIV vaccines.

## Introduction

The pandemic of acquired immune deficiency syndrome (AIDS), which is caused by human immunodeficiency virus type 1 (HIV-1), continues to be a serious challenge for global public health. Virus-specific CD8+ cytotoxic T lymphocyte (CTL) responses are crucial for controlling the infection of HIV-1 and simian immunodeficiency virus (SIV) (1–3), and therefore, candidates that are capable of inducing robust CTL responses are being widely developed for an effective HIV-1 vaccine. After termination of the highly anticipated STEP trial of Merck's adenovirus-vector HIV vaccine (4, 5), which was a major setback to the field of T cell-based HIV vaccine research, increasing findings in the era of post-STEP demonstrated that the next generation of T cell-based HIV vaccine candidates should enhance the quantity and quality of CTL responses to confer superior protective efficacy (6–8). Recently, a variety of vaccination regiments, including novel viral vector-based vaccines and heterologous prime/boost strategies, generated potent CTL responses and afforded significant protection against highly pathogenic SIV challenge in non-human primates, and several promising candidates are moving forward to preclinical and clinical trials (6, 9–13). As a result, it is of great interest to develop strategies to further enhance the magnitude, breadth, and polyfunctionality of HIV vaccine-elicited CD8+ cytotoxic T lymphocyte immune responses.

Immuno-inhibitory signal pathways are usually involved in the pathogenesis of tumor and infectious diseases, as well as in response to vaccination. The immune system is strongly activated during vaccination or viral infections, whereby high level expression of multiple immune-inhibitory receptors, such as programmed death 1 (PD-1, CD279), TIM-3, CD160, and LAG-3, are accompanied to balance the immune homeostasis (14–17). Among them, PD-1 signal pathway is extensively recognized as a promising target involved in the field of anti-tumor and anti-infection. PD-1, a member of the CD28 gene family, is a negative immunoregulatory molecule expressed on the cell surface of activated T, B, and myeloid lineage cells. The upregulated PD-1 expression resulted in suppression of cellular proliferation, cytotoxic function and anti-viral cytokine secretion, so that lead to exhaustion and anergy of cytotoxic T lymphocytes (CTLs). During HIV-1/SIV infection, the frequency of PD-1 expression on NK cells and HIV/SIV-specific T cells was associated with T cell dysfunction, viremia and AIDS disease progression (18–21). In addition, high level expression of PD-1 on memory CD4+ cells was also reported to be linked with HIV latency (22, 23). Moreover, studies have shown that blocking PD-1 signal pathway with anti-PD-L1 antibody in SIV-infected macaques and HIV-infected patients rebuilt the immune system, restored the function of specific T cells, and increased HIV-specific CD4 T cell proliferation (24–26). However, the importance of blocking PD-1 inhibitory pathway in modulating T cell functions during preventive vaccination against SIV infection in non-human primates is not clear.

In this study, we evaluated the immunomodulatory functions of SIV-specific immune responses by an anti-human PD-1 monoclonal antibody (named genolimzumab Injection) using *in vitro* and *in vivo* rhesus macaque models. We observed persistent stronger SIV-specific immune responses in rhesus monkeys immunized with SIV vaccine and genolimzumab compared to monkeys immunized with SIV vaccine only. Furthermore, immunized monkeys were challenged intrarectally with a highly pathogenic SIVmac239, and our data demonstrated that the regimen of SIV vaccine combined with genolimzumab achieved potent protection against SIV infection. These results of PD-1 blockade during vaccination in non-human primates provide new insights for the development of HIV vaccines.

## Materials and methods

### Antibodies and vaccines

Anti-human PD-1 monoclonal antibody (genolimzumab injection) was obtained from Genor Biopharma Co. Ltd (Pudpng, Shanghai, China), which is a subsidiary company for Walvax Biotechnology Co. Ltd (Walvax: 300142). This product has obtained approval for clinical trials by both China Food and Drug Administration (2016L10520) and Australia Therapeutic Goods Administration (NCT03053466).

Non-replicating adenoviruses (Ad-SIVgpe) expressing the SIVmac239 Gag, Pol and Env structural proteins were derived from our laboratory. The transgene in this recombinant adenovirus is driven by the CMV promotor. Construction, amplification, purification, identification and TCID50 assay for these recombinant adenoviruses are the same as our previously published methods (10).

SIVmac239-specific peptides were obtained through the NIH AIDS Research and Reference Reagent Program, and represented the entire amino acid sequences of Gag, Pol, Env, Nef, Vif, Vpx, Vpr, Rev, and Tat. These peptides are 15 amino acids in length with a shift of 11 overlapping residues. SIV peptides were dissolved in DMSO and peptides from a single antigen were pooled to a final concentration of 0.4 mg/peptide/ml.

### Characteristics of anti-PD-1 monoclonal antibody (genolimzumab injection)

To test the specificity of binding monkeys' PD-1, genolimzumab antibody and CD279 (PD-1)-PE eFlour 610 monoclonal antibody (Clone: eBioJ105, eBioscience) were mixed at a proportion of 1:1, and then incubated with peripheral blood mononuclear cells (PBMCs) from Chinese rhesus monkeys. Samples were analyzed with BD LSRFortessa^TM^ (BD Biosciences) and FlowJo software (Tree Star, Inc). Western blotting analysis was also performed to examine the reactivity of this antibody. The extracts of purified CD8+ T cells from monkey PBMC were subjected to SDS-PAGE and then transferred onto a PVDF membrane (Bio-Rad, Hercules, CA). After blocking for 1 h with 5% skim milk in PBS buffer, the membrane was incubated with genolimzumab antibody for 2 h. The membrane was washed and then incubated with horseradish peroxidase-conjugated goat anti-human IgG antibody or goat anti-mice IgG antibody at a 1:5,000 dilution for 1 h. Finally, the specific bands were visualized using enhanced chemiluminescence regents (ECL, GE Amersham). Anti-GAPDH antibody was used as internal loading control.

### Assays for evaluating cellular immune responses

The cellular immune assays including IFN-γ ELISPOT for total cellular immune responses, multi-color intracellular cytokine staining (ICS) for poly-functionality T lymphocytes, and carboxyfluorescein diacetate succinimidyl ester (CFSE) staining for *ex vivo* T cell proliferation were conducted in this study as our previously described (27). Briefly, for IFN-γ ELISPOT, freshly isolated PBMCs were added at 4 × 10^5^ cells/well in anti-monkey IFN-γ monoclonal antibody (BD Pharmingen) pre-coated 96-well plates containing Immobilon-P membrane (Millipore, USA). SIV peptide pools were added into cells for 20–24 h for stimulation, and then a polyclonal anti-monkey IFN-γ biotinylated detector antibody (BD Pharmingen) was added. The next day, the plates were washed and color was developed by incubating in NBT/BCIP (Pierce, Rockford, IL) for 10 min. Spots were counted under an ELISPOT reader (Bioreader 4000, BIOSYS, Germany), and data were reported as the number of spot-forming cells (SFC) per million PBMCs. Concanavalin A stimulation was used as a positive control in the ELISPOT assay.

For Multi-color ICS (intracellular cytokines staining) assays, one million cells were stimulated with SIV peptides for 2 h, and then Brefeldin A (BD Biosciences) was added for an additional 16 h. The cells were then washed and stained for 30 min with anti-CD3–Pacific Blue, anti-CD4–PE-CF594, anti-CD8–allophycocyanin (APC)–Cy7, anti-CD28–fluorescein isothiocyanate (FITC), and anti-CD95–phycoerythrin (PE)–Cy5. Next, the cells were suspended in 250 μl of Cytofix/Cytoperm solution (BD Pharmingen) for 20 min, washed with Perm/Wash solution (BD Pharmingen), and intracellularly stained with anti-IFN-γ-PE, anti-TNF-α-PE–Cy7 and anti-IL2-APC (BD Pharmingen) for 30 min. Samples were analyzed with BD LSRFortessaTM (BD Biosciences) instrument using the FlowJo software (Tree Star, Inc). PMA/I (Phorbol myristate acetate+ Ionomycin) stimulation was used as a positive control in the ICS assay.

For CFSE staining, PBMCs were incubated with 0.25 μM CFSE (Molecular Probes) at 37°C for 10 min, and then stimulated with SIV peptides. On day 6, cells were harvested and stained with anti-CD3-Pacific Blue (BD Biosciences), anti-CD4-allophycocyanin (APC, BD Biosciences), and CD8-APC-cy7 (BD Biosciences). Samples were analyzed with BD LSRFortessaTM (BD Biosciences) instrument using FlowJo software (Tree Star, Inc). Antigen-specific proliferating fraction in response to antigen was calculated by subtracting the proportion of proliferating cells in unstimulated samples. PMA/I stimulation was used as a positive control in the CFSE assay.

### Modulating T cell immune responses by PD-1 blockade *in vitro*

PBMCs were isolated from monkeys and then incubated with or without genolimzumab antibody at a concentration of 1–100 μg/ml. Next, cellular immune assays including IFN-γ ELISPOT, multi-color ICS, and CFSE-based proliferation were performed as described above.

### Animals and ethical statement

**Figure 1:**
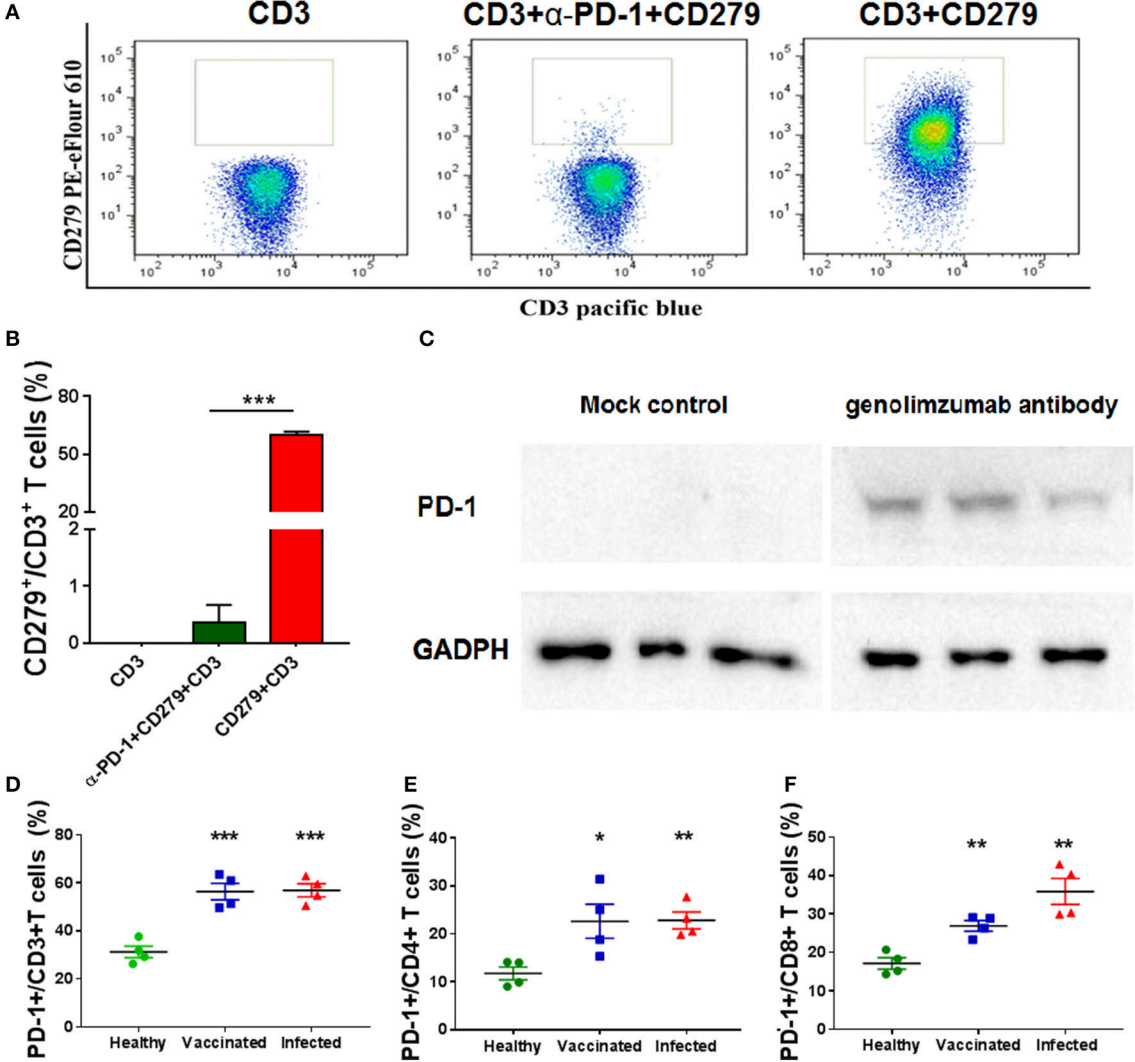
Characteristics of the anti-PD-1 monoclonal antibody used in this study. Anti-PD-1 antibody (genolimzumab) and CD279-PE eFlour 610 monoclonal antibody (known to be specific to recognize monkey PD-1) were mixed at the proportion of 1:1, and then incubated with peripheral blood mononuclear cells (PBMCs) from Chinese rhesus monkeys. Samples were analyzed with BD LSRFortessa. **(A)** Representative graphics of FACS analysis. **(B)** Statistical analysis of competitive experiment for genolimzumab and fluorescent-labeled anti-CD279 antibody. **(C)** The extracts of purified CD8+ T cells from monkey PBMC were subjected to SDS-PAGE and western blotting for assessing the reactivity between genolimzumab and monkey PD-1 protein. Mock control represented to perform same protocol but without incubation of genolimzumab. Anti-GAPDH antibody was used as internal loading control. PD-1 expression was significantly upregulated on the surface of CD3+ T cells **(D)**, CD4+ T cells **(E)** and CD8+ T **(F)** in monkeys infected with SIV and immunized with SIV vaccine. The final data are represented as the mean ± SEM from at least triplicate experiments. ^*^P < 0.05, ^**^P < 0.01, ^***^P < 0.001.

**Figure 2:**
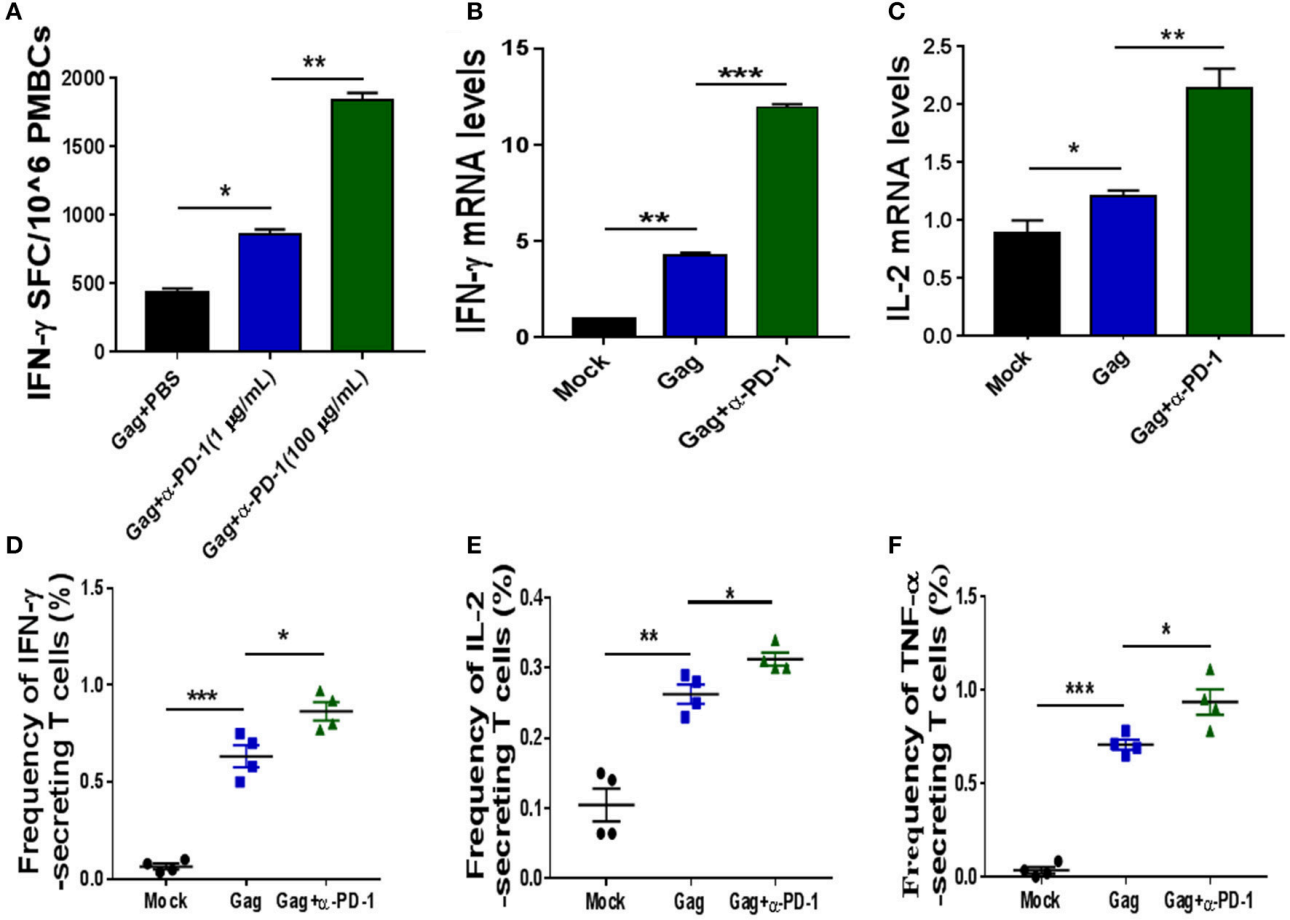
Modulating SIV-specific T cell immune responses by PD-1 blockade *in vitro*. SIV-specific T cells from chronic SIV-infected monkeys were stimulated with either SIV peptide pool alone or with SIV peptides in combination with genolimzumab, and then detected by IFN-γ-mediated enzyme-linked immunospot (ELISPOT) assay **(A)**. The above samples were also analyzed for IFN-γ expression **(B)** and IL-2 **(C)** expression by Q-PCR. Moreover, the frequency of SIV Gag-specific cytokine-secreting CD3+ T cells was further detected by intracellular cytokine staining. IFN-γ+ CD3+ T cells **(D)**, IL-2+CD3 T cells **(E)**, and TNF-α+CD3+ T cells **(F)** are indicated, respectively. The final data are represented as the mean ± SEM from at least triplicate experiments. ^*^P < 0.05, ^**^P < 0.01, ^***^P < 0.001.

**Figure 3:**
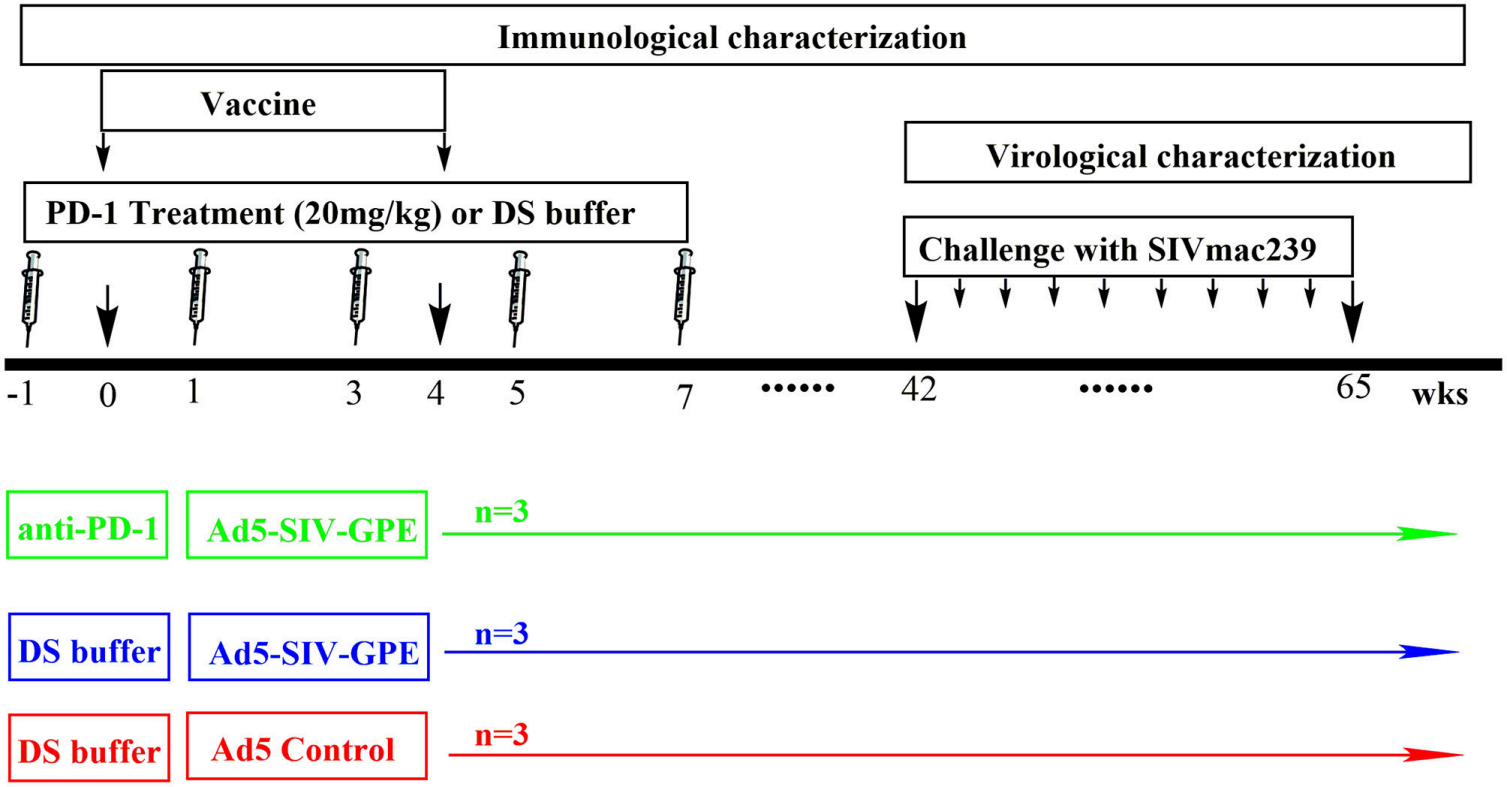
The schedule of vaccination and challenge for monkey experiment. Nine monkeys (4–6 years of age) that weighed 4–9 kg were randomly assigned into three groups: (1) three monkeys received genolimzumab injection (20 mg/kg in 1 ml DS buffer) through intravenous injection every 2 weeks, and rAd5-SIVgpe (10^11^ vp in 1 ml PBS buffer) through intramuscular injection at week 0 and week 4; (2) three monkeys received rAd-5SIVgpe (10^11^ vp in 1 ml PBS buffer) through intramuscular injection at week 0 and week 4; and (3) three monkeys received Ad5-empty as the mock group. At week 42 after the initial vaccination, animals were challenged intrarectally with repeated low-dosage of highly pathogenic SIVmac239. Viral load in plasma was monitored every week, and challenge was repeated every week in an incremental dosage ranging from 1 × 10^3^ to 1 × 10^5^ TCID_50_ until the animals were confirmed to be infected.

Chinese rhesus monkeys (*Macaca mulatta*) were housed in the experimental animal center of Guangzhou Institute of Biomedicine and Health (GIBH, Guangzhou, China). This study was carried out in accordance with the “Regulations for the Administration of Affairs Concerning Experimental Animals” by the State Council of People's Republic of China, and the protocol was approved by the Institutional Animal Care and Use Committee of GIBH (IACUC Permit Number: 2016004). All procedures were performed by trained personnel under the supervision of veterinarians. All animals were free of infection from simian immunodeficiency virus (SIV), simian T lymphotropic virus type 1 (STLV-1) and simian retrovirus (SRV) before the initiation of experiments. Twelve monkeys were used for *in vitro* experiments, which were in three settings of healthy, vaccinated and infected monkeys (Figures 1, 2). Four monkeys among them, which were involved in our previous studies (10, 27), were chronically infected with SIVmac239. The overall vaccination and challenge schedule is shown in Figure 3. Briefly, nine monkeys (4–6 years of age) that weighed 4–9 kg were randomly assigned into three groups: (1) three monkeys received PBS as the mock control group; (2) three monkeys received genolimzumab injection (20 mg/kg in 1 ml DS buffer) through intravenous injection every 2 weeks, and rAd5-SIVgpe (10^11^ vp in 1 ml PBS buffer) through intramuscular injection at week 0 and week 4; (3) three monkeys received rAd-5SIVgpe (10^11^ vp in 1 ml PBS buffer) through intramuscular injection at week 0 and week 4. At week 42 after the initial vaccination, animals were challenged intrarectally with repeated low-dosage challenge of SIVmac239 that is highly pathogenic, rhesus monkey-adapted, and neutralization-resistant. Viral load in plasma was monitored every week, and challenge was repeated every week in an incremental dosage ranging from 1 × 10^3^ TCID_50_ to 1 × 10^5^ TCID_50_ until the animals were confirmed to be successfully infected. Sequential peripheral blood samples were collected to evaluate the virological and immunological responses following the experiment schedule in Figure 3.

### Modulating T cell immune responses by PD-1 blockade *in vivo*

PBMCs were isolated from immunized monkeys, and then cellular immune assays including IFN-γ ELISPOT, multi-color ICS and CFSE-based proliferation were performed as described above. The numbers of circulating CD4+ and CD8+ T lymphocytes were determined by using Becton Dickinson Trucount tubes according to the manufacturer's instructions (BD Biosciences).

### Humoral immune assays

The SIV mac239-specific binding antibodies were detected by an enzyme-linked immunosorbent assay according to our described previously (27). The SIV mac239-specific neutralizing antibodies (nAb) were measured using a luciferase reporter gene assay in TZM-BL cells according to described previously (10).

### Assays for SIV viral RNA and DNA copies

The plasma samples were collected following standard protocols. Viral RNA was extracted from plasma using the QIAamp Viral RNA Mini kit (Qiagen) and quantified using the QuantiTect SYBR Green RT-PCR Kit (Qiagen) as described previously (27). The detection limits of assays were 100 copies per 1 ml plasma. SIV DNA copies in PBMCs were quantitated according to previously described methods (11). Briefly, PBMCs from the macaques were isolated by standard FicollHypaque density gradient centrifugation, and the total cellular DNA was isolated from 5 × 10^6^ cells using a QIAamp DNA Blood Mini kit (Qiagen). The SIV viral DNA was determined by a pair of primers specific to a conserved region of SIV gag gene. Quantitation was analyzed by comparing to the standard curve of SIV gag copies, and the β-actin gene was simultaneously amplified as a reference for input cell counts. PCR assays were performed with 200 ng samples of DNA.

### Data analysis

The flow cytometry data were analyzed using the Flowjo software, and graphical presentations were computed with the GraphPrism 5.01 software (GraphPad software Inc., La Jolla, CA, USA). The survival curves were analyzed by log-rank (Mantel-Cox) tests. Two-tailed *p*-values were calculated, and differences were considered as statistically significant when *P* < 0.05.

## Results

### Characteristics of anti-PD-1 monoclonal antibody (genolimzumab injection)

The anti-human PD-1 monoclonal antibody used in this study, named Genolimzumab Injection, is an IgG4 isotype. This antibody is expressed in a high level in a stable cell line of Chinese Hamster Ovary (CHO). The comprehensive results of *in vitro* bioactivity, *in vivo* pharmacology, pharmacokinetics and safety evaluation showed that genolimzumab had excellent druggability; therefore, this drug candidate is now being tested in clinical trials both in China (2016L10520) and Australia (NCT03053466). Since the following studies mainly use rhesus monkeys as a model, we firstly determine whether genolimzumab exerts cross-reactivity with rhesus monkey PD-1 proteins. Results showed that genolimzumab competitively interacted with a fluorescent-labeled anti-CD279 antibody (Figures 1A,B), which is specific to recognizing PD-1 molecules on the surface of monkey cells. This specific reactivity of monkey PD-1 protein was also confirmed by western blotting (Figure 1C). In addition, we observed that PD-1 expression was significantly upregulated on the surface of CD3+ T cells, CD4+ T cells, and CD8+ T cells in the monkeys infected with SIV and immunized with SIV vaccine (Figures 1D–F). To evaluate if genolimzumab exerts cell cytotoxicity, monkey PBMCs were treated with increasing concentrations of genolimzumab. Results showed no significant effect on cell viability at concentrations up to 100 μg/ml (Supplementary Figure 1). These findings demonstrated that genolimzumab was effectively bound to monkey PD-1, and this monoclonal antibody might be suitable for blockade of PD-1/PD-L1 signal pathways in SIV-infected or vaccinated monkeys.

### Modulating SIV-specific T cell immune responses by PD-1 blockade *in vitro*

We next investigated if genolimzumab antibodies block PD-1/PD-L1 inhibitory pathways and exert any effect on SIV-specific T cell immune responses. SIV-specific T cells in PBMCs, which were isolated from chronic SIV-infected monkeys, were stimulated with either SIV peptide pool alone or with SIV peptides in combination with genolimzumab, and then detected by IFN-γ-mediated enzyme-linked immunospot (ELISPOT) assay. Results showed that the frequency of SIV-specific IFN-γ-secreting cells was significantly increased when PBMCs were stimulated with SIV peptides in the presence of anti-PD-1 antibody (Figure 2A), as compared with PBMCs cultured with SIV peptides alone. The enhancement of SIV-specific T cell immune responses by PD-1 blockade was dose-dependent (Figure 2A). We also quantified the expression of IFN-γ (Figure 2B) and IL-2 (Figure 2C) by Q-PCR, and results were consistent with the ELISPOT assay. The enhancement of frequency of SIV-specific cytokine-secreting T cells by PD-1 blockage was further confirmed by intracellular cytokine staining, and the frequency of SIV-specific IFN-γ- (Figure 2D), TNF-α-(Figure 2E), and IL-2-(Figure 2F) secreting cells was significantly higher when PBMCs were stimulated with SIV peptides in the presence of anti-PD-1 antibody. Taken together, these results indicated that genolimzumab effectively blocked PD-1/PD-L1 inhibitory pathways and promoted SIV-specific T cell immune responses *in vitro*.

### Modulating SIV-specific immune responses by *in vivo* PD-1 blockade in rhesus monkeys

Next, to assess how this anti-PD-1 antibody affected SIV-specific immune responses *in vivo*, rhesus monkeys were intravenously injected with or without anti-PD-1 antibody every 2 weeks during SIV vaccine immunization scheduled as shown in Figure 3. The monkeys enrolled in this study were randomly assigned into three groups as described in Figure 3 and Supplementary Table 1. Based on pharmacokinetics study (Supplementary Figure 2), the interval administration of this antibody in this study was every 2 weeks to maintain sufficient concentrations in the plasma. In our study, all monkeys that received SIV vaccine in combination with genolimzumab showed good tolerance to this regimen with no observed adverse effects, based on routine blood biochemical examination and physical examination (Supplementary Table 2), implying that repeated administration of 20 mg/kg genolimzumab did not cause detectable toxicity in the monkeys.

**Figure 4:**
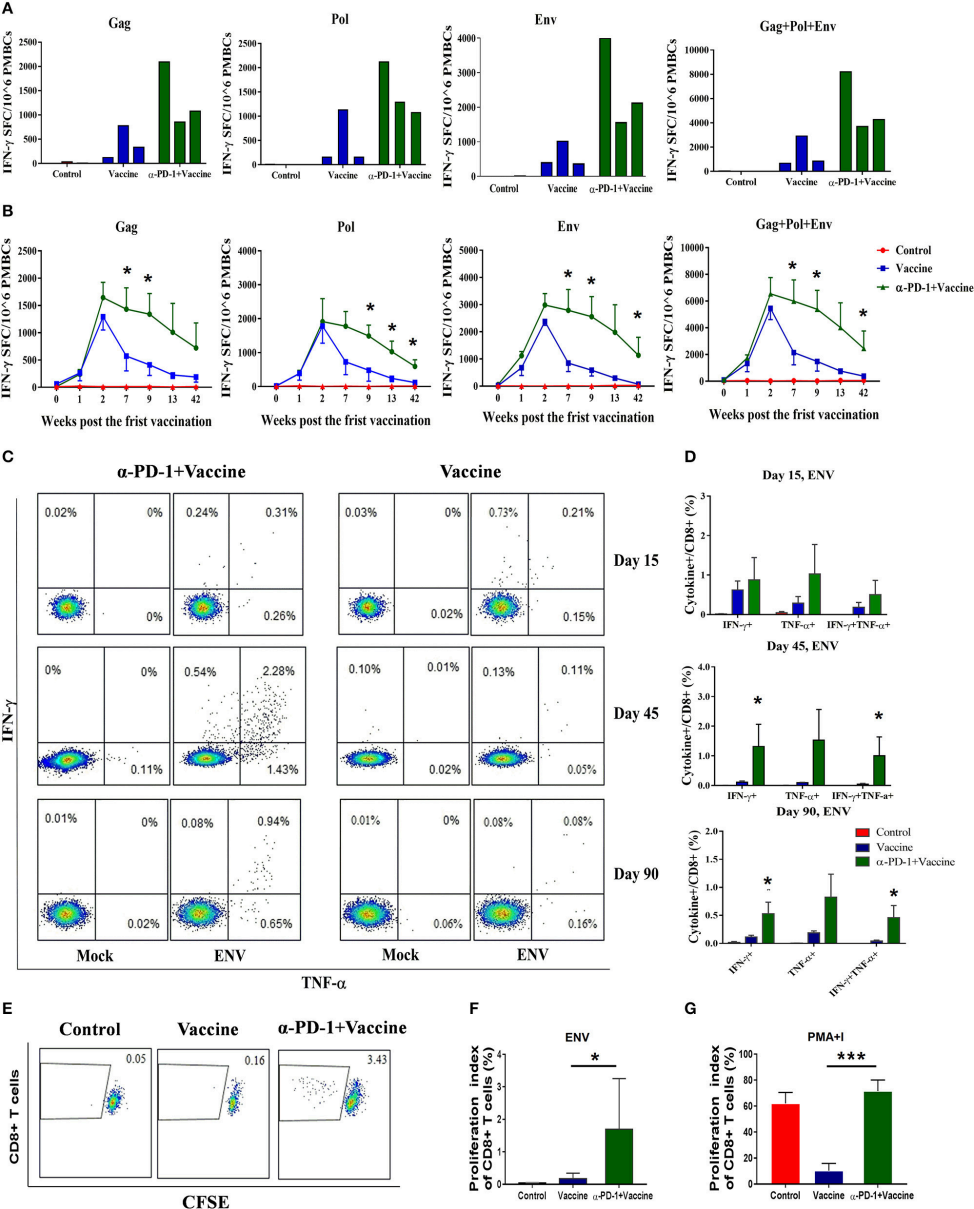
SIV-specific T cell immune responses regulated by PD-1 blockade *in vivo* in rhesus monkeys. Rhesus monkeys were intravenously injected with anti-PD-1 antibody during the SIV vaccine immunization schedule as described in Figure 3. For assessing total cellular immune responses induced by SIV vaccine regimen, IFN-γ-secreting cells were detected by ELISPOT assay over the course of immunization. **(A)** Representative data to illustrate IFN-γ-mediated ELISPOT cellular immune responses against SIV structure proteins (Gag, Pol, and Env), for each monkey at 9 weeks after first immunization. **(B)** Dynamic analysis of SIV-specific IFN-γ-mediated ELISPOT cellular immune responses over time until 42 weeks after first immunization. Data represent spot-forming cells (SFC) per million PBMCs. For assessing the polyfunctionality of SIV-specific CD8+ T cells, IFN-γ-and TNF-α-secreting cells were detected by intracellular cytokine staining (ICS) assays. **(C)** Representative flow cytometry dot plots for IFN-γ-and TNF-α-secreting CD8+ T cell populations. **(D)** Statistical analysis for the frequency of IFN-γ-and TNF-α-secreting CD8+ T cells by ICS. CFSE staining was used for assessing the ability of SIV antigen-stimulated T cell proliferation, whereby PBMCs were incubated with CFSE stain and stimulated with SIV peptides. **(E)** Representative dot plots for specific CD8+ T cell proliferation by CFSE staining. Statistical analysis for the proliferation index of CD8+ T cells in response to SIV antigen stimulation **(F)** or PMA+I simulation **(G)**. The daughter cells from proliferation had lower fluorescence intensity (CFSE-low cells). DMSO treatment was used as a negative control. ^*^P < 0.05; ^***^P < 0.001.

To evaluate SIV-specific cellular immune responses *in vivo*, IFN-γ-mediated ELISPOT assays were performed throughout the course of the experiment. Recombinant Ad-vectored vaccine expressing SIVmac239 Gag, Pol and Env structural proteins elicited potent immune responses in all immunized rhesus monkeys (Figure 4A). The frequency of positive responses against Gag, Pol, and Env peptides were detected at 1 week, and peaked at 2 week after priming immunization (Figure 4B and Supplementary Figure 3). We observed that responses in monkeys that received SIV vaccine in combination with genolimzumab were significantly higher than in monkeys from the vaccine-only group. This enhancement appeared at week 2 and was maintained over time for more than 42 weeks after priming immunization (Figure 4B, *p* < 0.05). In contrast, IFN-γ-mediated ELISPOT responses in the vaccine-only group declined over time, likely due to T cell exhaustion and apoptosis by extensive Ad vector-stimulated immune activation and PD-1 up-regulation (Figure 1D). Importantly, at time-point week 42 of initial viral challenge, the frequency of positive responses in the group of SIV vaccine combined with genolimzumab remained significantly higher than that in the vaccine-only group (Figure 4B). Furthermore, compared with the vaccine-only group, all animals in the SIV vaccine combined with genolimzumab group maintained significantly higher levels of polyfunctionality in SIV-specific CD8+ T cells, particularly for IFN-γ and TNF-α release (Figures 4C,D). Nevertheless, the ability of SIV antigen-stimulated T cell proliferation was also significantly improved by co-administration of genolimzumab (Figures 4E,F). The positive controls for ELISPOT, ICS, and CFSE assays were indicated in Supplementary Figure 4. Very interestingly, the ability of mitogen-stimulated nonspecific T cell proliferation was significantly decreased in the SIV vaccine-only group, but restored to normal level by co-administration of genolimzumab (Figure 4G). We also detected the change of central and effector T memory subsets (Tcm and Tem), based on CD95/CD28 marker as descripted in our previous study (10). After anti-PD-1 treatment, we did not observe a significant change of the proportions of Tcm and Tem. This might be due to large inter-individual variation. Taken together, these results showed that administration of genolimzumab significantly improved the magnitude, persistence, polyfunctionality, and proliferation of SIV-specific T cell immune responses *in vivo* in monkeys.

We also found relatively higher levels of binding antibodies in the SIV vaccine/anti-PD-1 group compared to vaccine-only group at the initial stages after immunization (week 2), suggesting that PD-1 blockade during vaccination facilitated in rapid induction of humoral immune responses. However, such differences disappeared over time, and this regimen of SIV vaccine/anti-PD-1 antibody induced a titer of SIVmac239-specific binding antibodies that was equivalent to the SIV vaccine-only regimen (Supplementary Figure 5). In addition, no detectable neutralizing antibodies against SIVmac239 were observed.

### Improved immune protection against SIVmac239 infection by *in vivo* PD-1 blockade in rhesus monkeys

**Figure 5:**
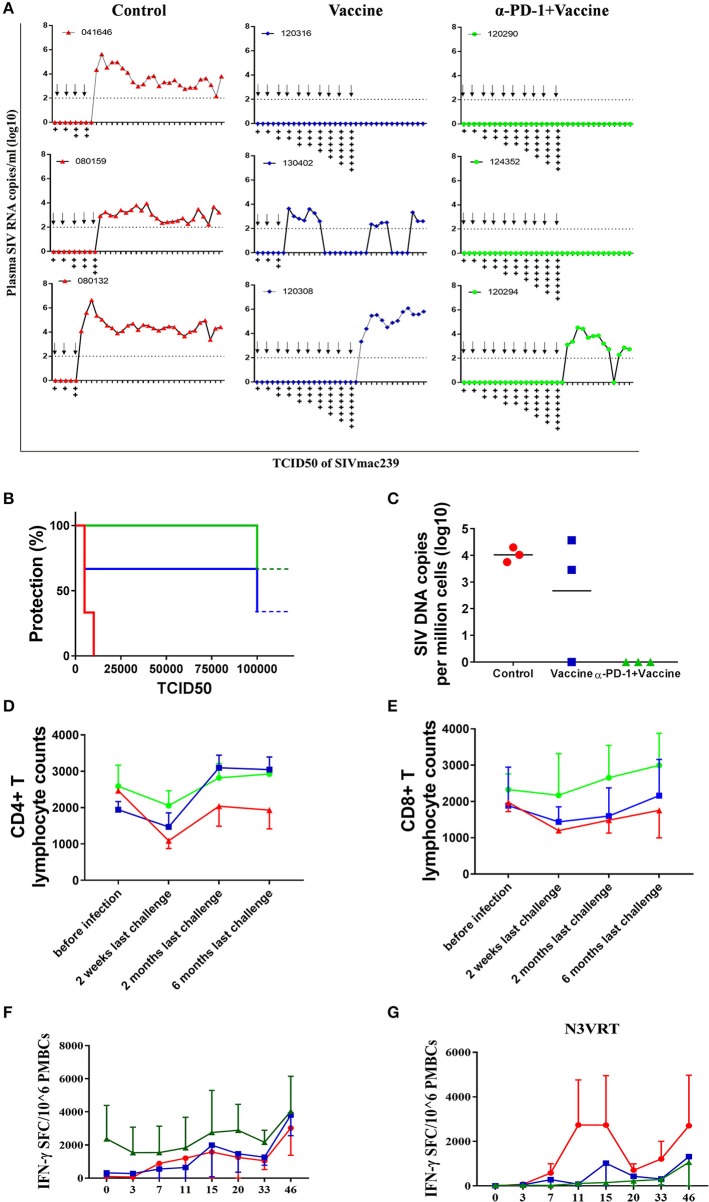
Immune protection against SIVmac239 infection by PD-1 blockade in rhesus monkeys. 42 weeks after first immunization, all monkeys were intrarectally challenged with highly pathogenic SIVmac239, at an incremental dosage ranging from 1,000 TCID_50_ (two times), 5,000 TCID_50_ (two times), 10,000 TCID_50_ (two times), 15,000 TCID_50_ (single time), 25,000 TCID_50_ (single time), 50,000 TCID_50_ (single time) to 100,000 TCID_50_ (single time). **(A)** Dynamic viral load in plasma for each animal was monitored over time by Q-PCR. Challenge was stopped when animal was confirmed to become infected.Arrows represent the time of SIV challenge. Symbol “+” represents the TCID_50_ of SIVmac239 challenge: +:1,000; + +:5,000; + + +:10,000; + + + +:15,000; + + + + +:25,000; + + + + + +:50,000; + + + + + + +:100,000. **(B)** Analysis of protection ratio under different doses of SIV challenge in different groups. All monkeys in the control group (3/3) and most in the vaccine-only group (2/3) became infected when challenged under a lower challenge dosage. In contrast, all animals that received the regimen of SIV vaccine combined with anti-PD1 antibody conferred full protection against SIVmac239 challenge up to 50,000 TCID_50_, although one monkey (#120294) became infected after challenged with an extremely high dose of 100,000 TCID_50_. **(C)** SIV viral DNA copies in PBMCs were assessed using PCR assay. The data showed the peak value of DNA copies after SIV infection. The number of CD4+ T cells **(D)** and CD8+ T cells **(E)** in monkeys' peripheral blood samples were monitored by using Trucount tubes. **(F)** For further assessment of immune protection, SIV-specific memory immune responses against antigens of Gag, Pol and Env were monitored over time post-infection by ELISPOT assay. **(G)** Immune responses against SIV accessory and regulatory proteins (Nef, Vif, Vpr, Vpx, Tat, and Rev) were also monitored over time after infection by ELISPOT assay. Data represents spot-forming cells (SFC) per million PBMCs.

We next sought to verify whether the superior immune responses elicited by the regimen of SIV vaccine combined with genolimzumab would confer better control of pathogenic SIVmac239 infection. Forty-two weeks after immunization, monkeys were challenged intrarectally with highly pathogenic SIVmac239 virus at an incremental dosage ranging from 1,000 to 100,000 TCID_50_ (Figure 5A). Viral load in plasma was monitored twice every week, and challenge was stopped when the animal was confirmed to be infected. Notably, all animals that received the regimen of SIV vaccine combined with genolimzumab conferred a full protection against nine challenges of SIVmac239 at dosed up to 50,000 TCID_50_, although one monkey (#120294) became infected after being challenged with an extreme high dose of 100,000 TCID_50_ (Figure 5B). In contrast, most monkeys in the control group (3/3) and vaccine-only group (2/3) became infected, even under a lower challenge dosage. For example, two individuals (#04164, #080132) in the control group and one (#130402) in the vaccine-only group became infected when challenged with 5,000 TCID_50_ of SIVmac239 (Figure 5B). Then, SIV viral DNA copies in PBMCs were assessed using PCR assay (Figure 5C). In all monkeys from the control group, viral DNA was detected within 1 week post-infection and persisted at a high level over time, reflecting the rapid integration into the host genome by this highly pathogenic SIVmac239 virus. In contrast, all of the protected monkeys that received vaccination and PD-1 antibody showed significant reductions of viral DNA to undetectable levels (Figure 5C), suggesting that our strategy led to substantial reduction of SIV infection and replication. We also observed that the number of CD4+ T cells transiently dropped at the initial infection stage and fluctuated in the subsequent days. There was a trend of the preservation of CD4+ T cells in protected monkeys (Figure 5D), though there was no statistical differences due to large inter-individual variation and limited number of animals. Consistent with the above data (Figure 4), administration of genolimzumab significantly improved the number of CD8+ T cells in monkeys (Figure 5E).

To further assess immune protection, SIV-specific immune responses were continually monitored after infection. Memory immune responses against SIV antigens of Gag, Pol, and Env were quickly boosted in response to SIV infection. In particular, the magnitude of positive responses in animals treated with SIV vaccine combined with genolimzumab were significantly higher than those detected in the vaccine-only and control groups (Figure 5F). Moreover, despite a decline after the peak responses, antigen-specific T cell responses remained relatively stable in the SIV vaccine combined with genolimzumab group throughout the subsequent phase of infection.

Another interesting observation in our study was that there were rapidly stronger SIV-specific immune responses against accessory and regulatory proteins (Nef, Tat, Rev, Vif, Vpr, and Vpx) in infected monkeys compared to those of vaccinated and uninfected animals (Figure 5G), reflecting the elevated responses in reply to the rise of viral replication in unprotected monkeys, which coincided with a rise in viral plasma load in these animals (Figure 5A). However, there was no detectable immune response against accessory and regulatory proteins in the uninfected animals, implying that a full protection was achieved by anamnestic immune responses against SIV antigens of Gag, Pol, and Env. Collectively, these findings suggested that SIV vaccine combined with genolimzumab regimens generated distinct immune response profiles and conferred better control of pathogenic SIVmac239 infection.

## Discussion

Vaccination is the most promising approach for eradicating infectious diseases, but to-date no HIV vaccine is available for clinical use, mostly because the immune correlates for protection are not fully understood (28–30). Both broadly neutralizing antibodies (bnAbs) and broad-spectrum cytotoxic T lymphocyte (CTL) responses play critical roles in controlling HIV-1 infection. Currently, vaccine candidates capable of eliciting bnAbs to neutralize diverse strains of HIV-1 remain an unsolved goal in HIV-1 research. A number of bnAbs against HIV-1 had been reported, and scientists are continually working to develop novel antigen designs that may induce bNAbs (31–34). However, these attempts have so far not demonstrated to be successful. Alternatively, it is of great interest to develop the next generation of T cell-based HIV vaccine, which is expected to elicit an enhanced magnitude, breadth and polyfunctionality of CD8+ cytotoxic T lymphocyte immune responses.

Studies have shown that virus-specific CD8+ cytotoxic T lymphocyte (CTL) responses are crucial for controlling HIV/SIV infection and replication (1–3). However, viral infection, especially chronic HIV-1/SIV infection, often cause the exhaustion and dysfunction of CD8+ T cells due to continuous antigenic stimulation (35–37). One of the features of exhaustion and dysfunction is the high level expression of multiple immune-inhibitory receptors such as programmed death 1 (PD-1), TIM-3, and CD160 (14, 23, 38). We confirmed that PD-1 expression was significantly upregulated on the surface of T cells not only in monkeys infected with SIV, but also in monkeys immunized with SIV vaccine (Figure 1D). Vaccination-induced PD-1 upregulation might give rise to compromise the persistent immunogenicity of the SIV vaccine. A number of studies supported that the increased PD-1 expression and subsequent engagement to its ligands (PD-L1 and PD-L2) triggered host immune system in a state of immune suppression, characterized by the impairment and exhaustion of T cells with reduced activation, proliferation, and cytokine secretion (23, 39–42). Moreover, the dysfunctional and ineffective T cell responses led to the inability of the host to clear the persisting pathogen, and greatly exacerbated the establishment of chronic viral infection and disease progression, such as in HIV, hepatitis B virus (HBV), and hepatitis C virus (HCV) infections in humans (41, 43–45).

As such, blockade of the PD-1 signal pathway could rebuild the immune system and restore the function of exhausted CD8+ T cells. Actually, previous studies indicated that PD-1 blockade resulted in enhanced SIV-Specific CD8+ T cells with improved functional quality (46), although how PD-1 blockade affects the HIV/SIV replication and infection remains controversial. In a therapeutic model of SIV-infected monkeys, it was showed that the improved immunity by PD-1 blockade afforded a significant reduction in SIV viral load and prolonged the survival of SIV-infected macaques (46). However, another study indicated that viral loads in the chronic SIV-infection rhesus model increased transiently after treatment with infusion of anti-PD-1 antibody, peaking at 5 days and returning to initial levels 19 days later (47). For prophylactic vaccination, PD-1 blockade generated an enhanced HIV-1 Gag-specific CD8+ immune response following dendritic cell-directed immunization, and this enhanced cellular immune response was correlated with improved viral control against Gag-expressing vaccinia virus challenge in mice (25). To our knowledge, little information has been reported related to how PD-1 blockade affects the immunogenicity and protection of prophylactic SIV vaccine in non-human primate models.

In this study, we therefore evaluated the immunomodulatory functions for SIV-specific immune responses by an anti-human PD-1 monoclonal antibody using *in vitro* and *in vivo* rhesus macaque models. Cohorts of Chinese rhesus monkeys were immunized by SIV vaccine with or without anti-human PD-1 monoclonal antibody. All of the monkeys enrolled in this study have been distributed carefully based on weight, age and genotype, and all of them were Mamu-A1^*^01-negative, which is a previously reported protective allele (48, 49). Considering only three animals per group were used in this pilot study of rhesus macaques, only females were used in order to minimize the possible impact of gender factors. Our future work will expand the scale of animals, and more factors, including a balanced gender ratio, will be taken into account to avoid bias of vaccine efficacy. Another limitation of this study is that we only analyzed the immunological and virological changes in the blood, and thus future work should consider including tissue biopsies to check the local T cellular responses at the site of SIV transmission (e.g., rectal).

Taken together, our results demonstrated that co-administration of anti-PD-1 monoclonal antibody significantly improved the magnitude, persistence, polyfunctionality and proliferation of vaccination-elicited, SIV antigen-specific T cell immune responses induced *in vivo* in rhesus monkeys. More importantly, the superior immune responses conferred a better control of pathogenic SIVmac239 infection. These results of PD-1 blockade during prophylactic vaccination in non-human primates provide new insight for the development of HIV vaccines. Our further research will apply the present findings to prospective preclinical and clinical studies.

## Author contributions

CS and LC conceived and designed the experiments. EP, FF, PL, QY, XM, CW, and JZ performed the experiments. EP and CS analyzed the data. RC and HY provided reagents. CS wrote the manuscript. All of the authors read the final manuscript.

### Conflict of interest statement

RC and HY are employed by Genor Biopharma Co. Ltd., Shanghai. The remaining authors declare that the research was conducted in the absence of any commercial or financial relationships that could be construed as a potential conflict of interest.

## Supplementary material

Supplementary Table 1 Summary of Chinese rhesus macaques used in this study.

Supplementary Table 2 Blood biochemical examination results for monkeys used in this study.

Supplementary Figure 1 Cytotoxicity on rhesus macaque PBMCs by anti-PD-1 antibody treatment. Monkey PBMCs were incubated with anti-PD-1 antibody at the given concentrations for 24 h, and then cell viability was measured by CCK-8 kit. The final data are represented as the mean ± SEM from at least three independent experiments.

Supplementary Figure 2 Pharmacokinetics parameter of anti-PD-1 monoclonal antibody used in this study. Thirty-two cynomolgus monkey received different doses of anti-PD-1 antibody (2, 5, or 50 mg/kg, *n* = 8 individuals for each dose) through intravenous injection at day 0, and then residual anti-PD-1 antibody levels in plasma were monitored by ELISA until 1,008 h after injection.

Supplementary Figure 3 SIV-specific IFN-γ-secreting T cell immune responses in each rhesus monkey. Rhesus monkeys were immunized and detected as described in Figures 3, 4. IFN-γ-mediated ELISPOT cellular immune responses against SIV Gag **(A)**, Pol **(B)**, and Env **(C)** were monitored for each monkey over time until 42 weeks post-immunization. Data represents spot-forming cells (SFC) per million PBMCs.

Supplementary Figure 4 The positive controls for ELISPOT, ICS and CFSE assays in this study. **(A)** 10^5^ PBMCs/well were performed as described in above methods of ELISPOT assay, but ConA (1 μg/ml) instead of SIV peptide pools was added into cells as positive stimulation. **(B)** One million of monkey PBMCs were stimulated with a cocktail of PMA (8 ng/ml) and ionomycin (200 ng/ml) for 2 h, and then processed as described in above methods of ICS assay. Representative plots were depicted to analyze the frequency of cytokines-positive T cells. **(C)** One million of monkey PBMCs were labeled with CFSE, and then cultured for 5 days with a cocktail of PMA (8 ng/ml) and ionomycin (200 ng/ml) as positive stimulators. The ability of T lymphocyte proliferation was analyzed by flow cytometry.

Supplementary Figure 5 SIV-specific binding antibodies in rhesus monkeys. The SIV mac239-specific binding antibodies were detected using previously described methods. The final data are represented as the mean ± SEM. ^*^*P* < 0.05.
