# Supplementary material for: Immune Protection of SIV Challenge by PD-1 Blockade During Vaccination in Rhesus Monkeys
Source: Front Immunol. 2018 Oct 23;9:2415. doi: 10.3389/fimmu.2018.02415 (PMC6206945; doi:10.3389/fimmu.2018.02415)
Supplement: Supplementary Table 1 — Summary of Chinese rhesus macaques used in this study. [file Data_Sheet_1.docx]

**Supplementary Table 1. Summary of Chinese rhesus macaques used in this study**

| **Group** | **Number** | **ID** | **Sex** | **Weight/kg** | **Immunogen** |
| --- | --- | --- | --- | --- | --- |
| Vaccine+PD-1 | PD-SIV-1 | 120290 | Female | 5.45 | Ad5-SIV-GPE |
|  | PD-SIV-2 | 120294 | Female | 4.74 | Ad5-SIV-GPE |
|  | PD-SIV-3 | 124352 | Female | 6.06 | Ad5-SIV-GPE |
| Vaccine | PD-SIV-4 | 120308 | Female | 5.50 | Ad5-SIV-GPE |
|  | PD-SIV-5 | 130402 | Female | 5.81 | Ad5-SIV-GPE |
|  | PD-SIV-6 | 120316 | Female | 4.48 | Ad5-SIV-GPE |
| Control | PD-SIV-7 | 041646 | Female | 6.15 | mock |
|  | PD-SIV-8 | 080159 | Female | 10.40 | mock |
|  | PD-SIV-9 | 080132 | Female | 6.90 | mock |

**Supplementary Table 2. Blood biochemical examination results for monkeys used in this study**

| **Group** | **ID** | **Date** | **ALT** | **AST** | **BUN** | **CRE** | **LDH** |
| --- | --- | --- | --- | --- | --- | --- | --- |
| Vaccine+PD-1 | 120290 | Before  PD-1 antibody | 39.7 | 72.5 | 21.0 | 51.0 | 668.8 |
|  |  | 1 day after  PD-1 antibody | 45.4 | 61.1 | 7.4 | 52.4 | 443.8 |
|  |  | 1 weekafter  PD-1 antibody | 25.0 | 69.4 | 7.4 | 59.3 | 672.2 |
|  |  | 1 month after PD-1 antibody | 24.0 | 53.8 | 9.2 | 65.7 | 332.1 |
|  | 120294 | Before  PD-1 antibody | 71.4 | 82.4 | 5.6 | 55.3 | 805.5 |
|  |  | 1 day after  PD-1 antibody | 66.5 | 65.4 | 6.7 | 45.1 | 463.9 |
|  |  | 1 week after  PD-1 antibody | 58.0 | 87.9 | 6.6 | 60.0 | 419.5 |
|  |  | 1 month after PD-1 antibody | 55.0 | 56.0 | 10.6 | 81.5 | 453.7 |
|  | 124352 | Before  PD-1 antibody | 50.1 | 47.2 | 5.4 | 43.5 | 367.4 |
|  |  | 1 day after  PD-1 antibody | 33.6 | 39.9 | 5.7 | 43.3 | 226.6 |
|  |  | 1 week after  PD-1 antibody | 26.0 | 58.7 | 4.8 | 45.9 | 390.5 |
|  |  | 1 month after PD-1 antibody | 43.0 | 42.1 | 5.9 | 63.8 | 274.6 |
| Vaccine | 120308 | Before  PD-1 antibody | 31.7 | 71.2 | 5.4 | 44.1 | 802.3 |
|  |  | 1 day after  PD-1 antibody | 34.0 | 46.6 | 5.6 | 44.5 | 435.6 |
|  |  | 1 week after  PD-1 antibody | 32.6 | 24.9 | 5.5 | 56.1 | 521.0 |
|  |  | 1 month after PD-1 antibody | 25.0 | 39.5 | 5.3 | 62.1 | 298.7 |
|  | 130402 | Before  PD-1 antibody | 50.8 | 65.5 | 6.8 | 40.8 | 471.2 |
|  |  | 1 day after  PD-1 antibody | 65.5 | 54.8 | 7.4 | 41.4 | 314.7 |
|  |  | 1 week after  PD-1 antibody | 37.0 | 70.1 | 6.0 | 51.0 | 304.0 |
|  |  | 1 month after PD-1 antibody | 26.0 | 45.7 | 2.4 | 58.8 | 233.7 |
|  | 120316 | Before  PD-1 antibody | 62.0 | 72.4 | 7.2 | 42.0 | 517.3 |
|  |  | 1 day after  PD-1 antibody | 56.7 | 57.1 | 7.4 | 42.3 | 388.9 |
|  |  | 1 week after  PD-1 antibody | 48.0 | 70.4 | 7.2 | 46.0 | 667.0 |
|  |  | 1 month after PD-1 antibody | 39.0 | 242.5 | 8.2 | 51.3 | 432.4 |
| Control | 041646 | Before  experiment | 72.2 | 35.2 | 8.2 | 71.7 | 568.5 |
|  |  | 1 day after  mock treatment | 77.1 | 52.8 | 6.2 | 41.5 | 620.0 |
|  |  | 1 week after  mock treatment | 35.7 | 32.8 | 7.5 | 71.2 | 652.4 |
|  |  | 1 month after mock treatment | 65.4 | 52.2 | 5.5 | 45.5 | 686.5 |
|  | 080159 | Before  experiment | 30.0 | 32.2 | 7.0 | 58.1 | 585.4 |
|  |  | 1 day after  mock treatment | 27.8 | 44.0 | 6.4 | 56.3 | 638.2 |
|  |  | 1 week after  mock treatment | 57.9 | 37.5 | 6.5 | 54.1 | 583.6 |
|  |  | 1 month after mock treatment | 34.6 | 42.1 | 6.4 | 53.6 | 888.2 |
|  | 080132 | Before  experiment | 77.7 | 64.2 | 11.2 | 67.5 | 892.4 |
|  |  | 1 day after  mock treatment | 68.2 | 40.6 | 5.7 | 42.7 | 597.6 |
|  |  | 1 week after  mock treatment | 23.2 | 34.7 | 5.6 | 51.7 | 665.2 |
|  |  | 1 month after mock treatment | 44.9 | 44.7 | 6.8 | 41.7 | 447.6 |

**Note: ALT:  alanine transaminase; AST: aspartate aminotransferase; BUN: blood urea nitrogen ; CRE: creatinine; LDH: lactate dehydrogenase.**
