# Supplementary figures and images for: Immune Protection of SIV Challenge by PD-1 Blockade During Vaccination in Rhesus Monkeys
Source: Front Immunol. 2018 Oct 23;9:2415. doi: 10.3389/fimmu.2018.02415 (PMC6206945; doi:10.3389/fimmu.2018.02415)

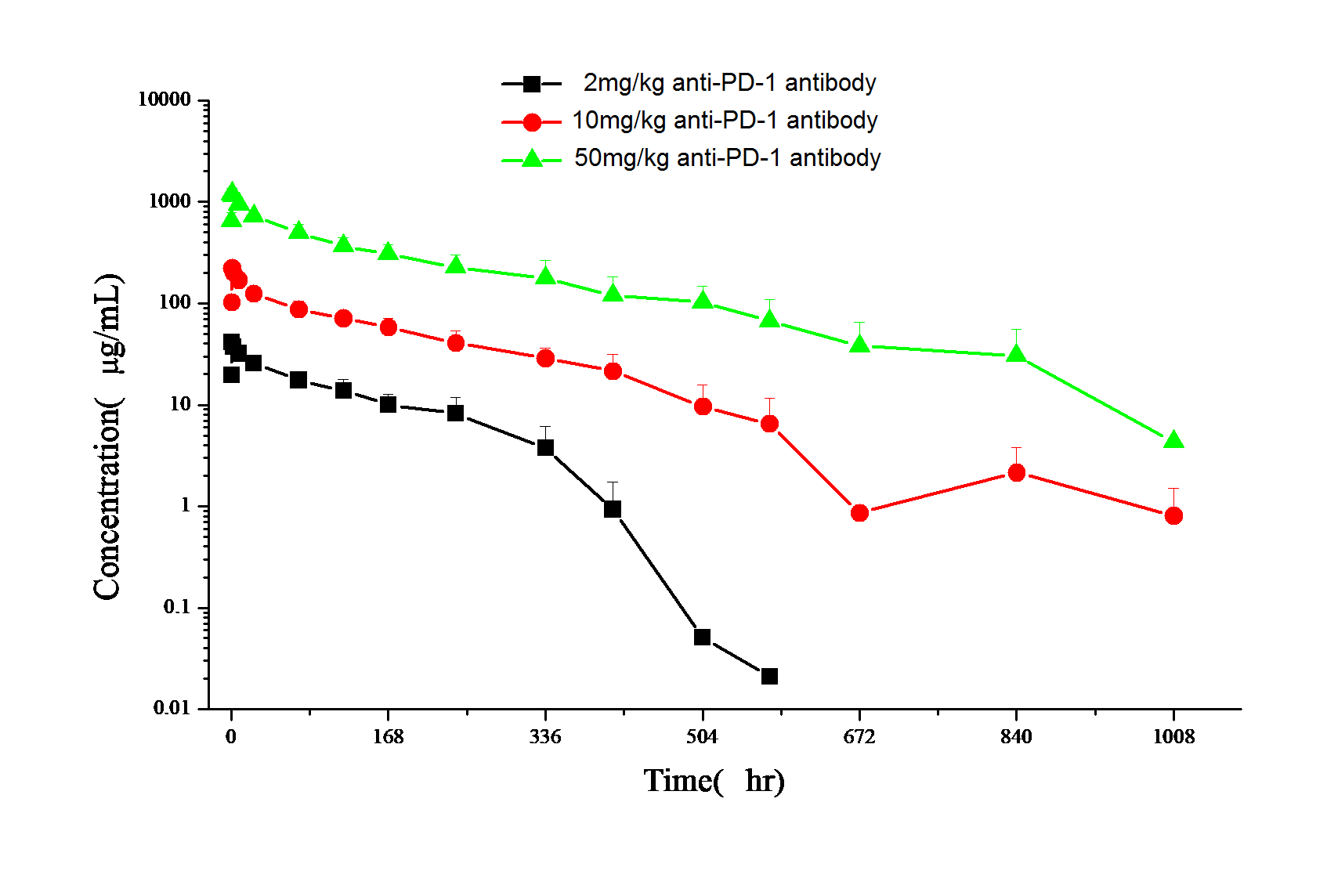

Supplement: Supplementary Figure 2 — Pharmacokinetics parameter of anti-PD-1 monoclonal antibody used in this study. Thirty-two cynomolgus monkey received different doses of anti-PD-1 antibody (2, 5, or 50 mg/kg, n = 8 individuals for each dose) through intravenous injection at day 0, and then residual anti-PD-1 antibody levels in plasma were monitored by ELISA until 1,008 h after injection. [file Image_2.TIF]

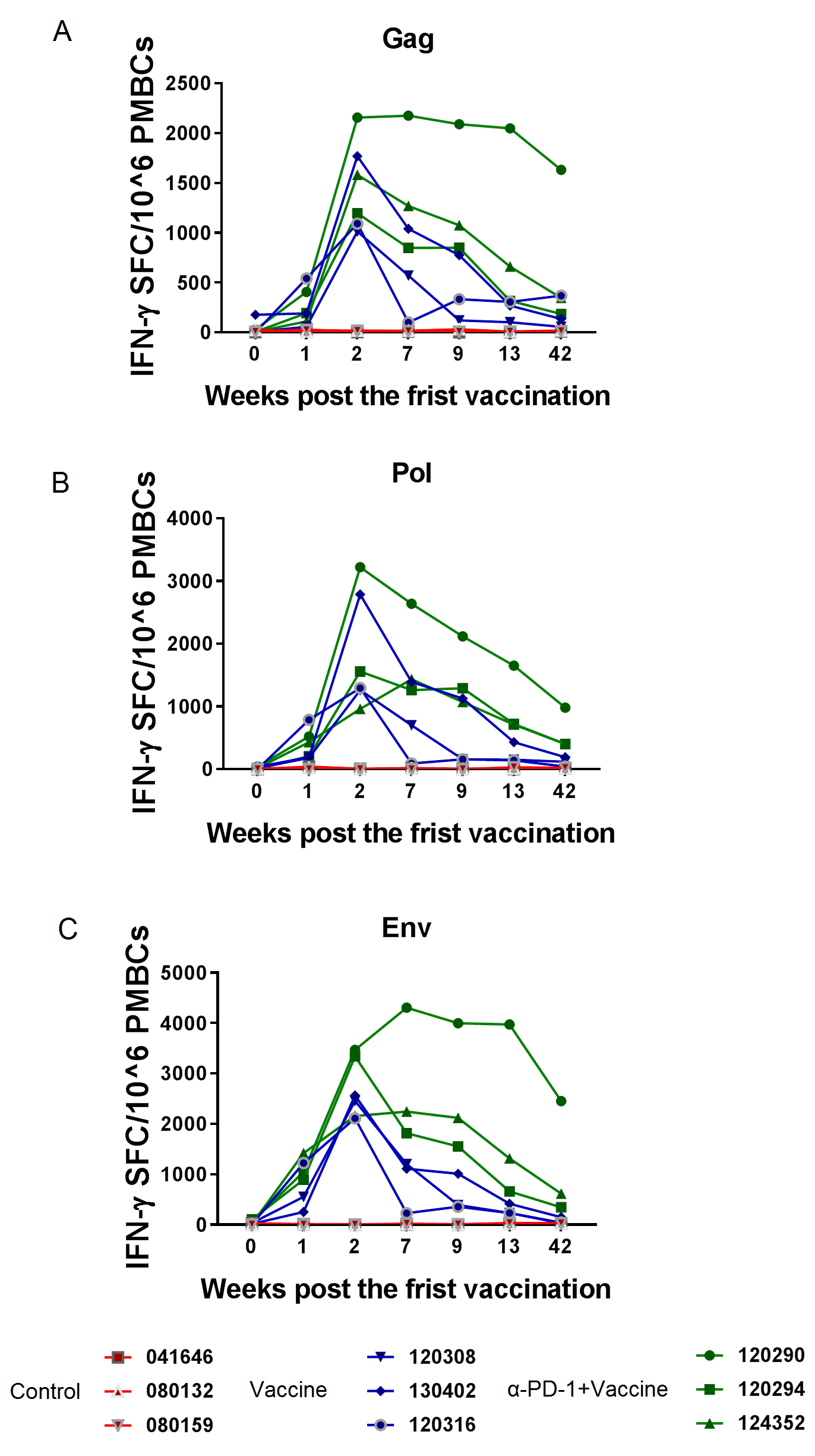

Supplement: Supplementary Figure 3 — SIV-specific IFN-γ-secreting T cell immune responses in each rhesus monkey. Rhesus monkeys were immunized and detected as described in Figures 3, 4. IFN-γ-mediated ELISPOT cellular immune responses against SIV Gag (A), Pol (B), and Env (C) were monitored for each monkey over time until 42 weeks post-immunization. Data represents spot-forming cells (SFC) per million PBMCs. [file Image_3.TIF]

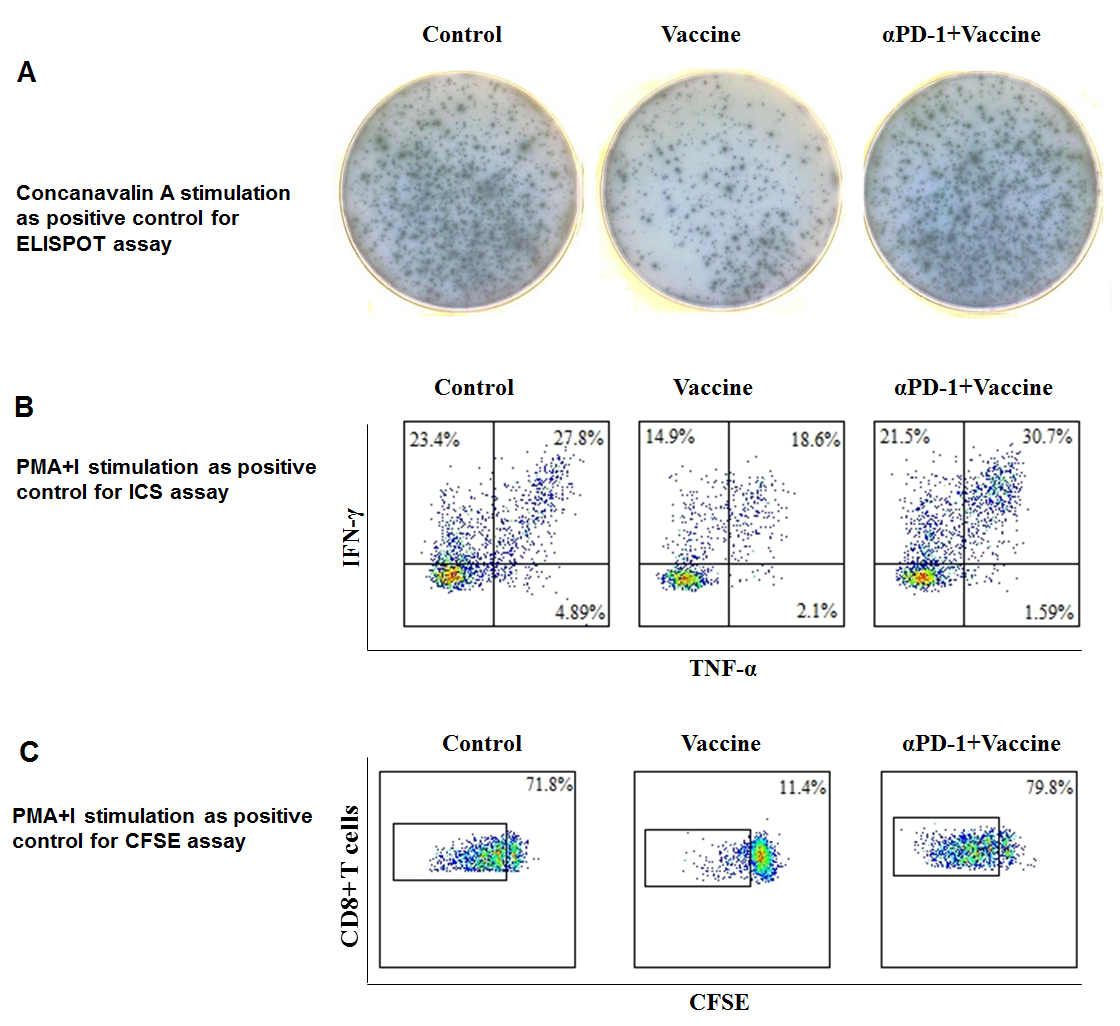

Supplement: Supplementary Figure 4 — The positive controls for ELISPOT, ICS and CFSE assays in this study. (A) 105 PBMCs/well were performed as described in above methods of ELISPOT assay, but ConA (1 μg/ml) instead of SIV peptide pools was added into cells as positive stimulation. (B) One million of monkey PBMCs were stimulated with a cocktail of PMA (8 ng/ml) and ionomycin (200 ng/ml) for 2 h, and then processed as described in above methods of ICS assay. Representative plots were depicted to analyze the frequency of cytokines-positive T cells. (C) One million of monkey PBMCs were labeled with CFSE, and then cultured for 5 days with a cocktail of PMA (8 ng/ml) and ionomycin (200 ng/ml) as positive stimulators. The ability of T lymphocyte proliferation was analyzed by flow cytometry. [file Image_4.TIF]

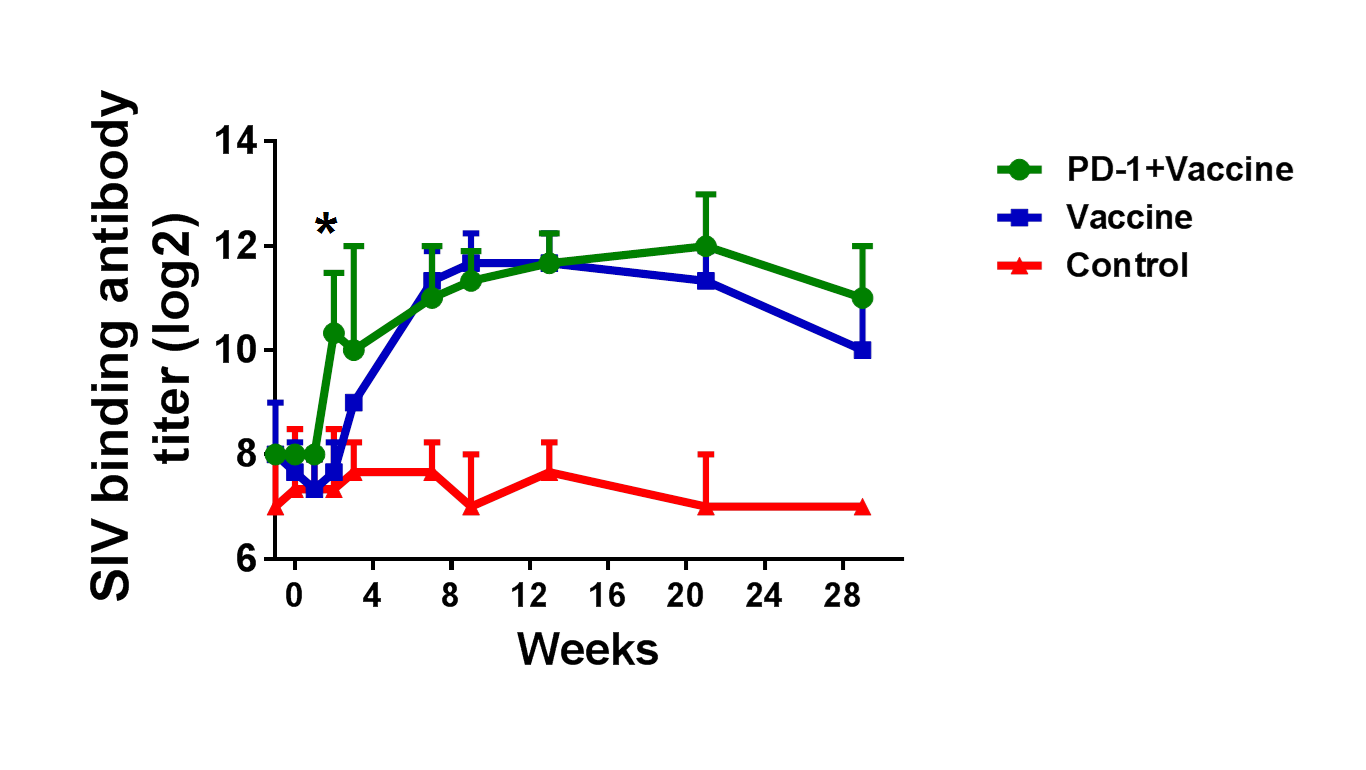

Supplement: Supplementary Figure 5 — SIV-specific binding antibodies in rhesus monkeys. The SIV mac239-specific binding antibodies were detected using previously described methods. The final data are represented as the mean ± SEM. *P < 0.05. [file Image_5.TIF]
